# Treatment of refractory uveitic macular edema with dexamethasone intravitreal implants in a pediatric patient with bilateral granulomatous idiopathic panuveitis: a case report

**DOI:** 10.1186/1869-5760-3-61

**Published:** 2013-10-22

**Authors:** Serge Bourgault, Maryam Aroichane, Leah A Wittenberg, Andréane Lavallée, Patrick E Ma

**Affiliations:** 1 Department of Ophthalmology and Visual Sciences, University of British Columbia, Vancouver, British Columbia V5Z 3N9, Canada; 2 Département d’Ophtalmologie et ORL - Chirurgie cervico-faciale, Université Laval, Québec, Québec G1V 0A6, Canada; 3 Department of Ophthalmology and Visual Sciences, British Columbia Children’s Hospital, University of British Columbia, Vancouver, British Columbia V6H 3N1, Canada

**Keywords:** Dexamethasone intravitreal implant, Ozurdex^®^, Cystoid macular edema, Uveitic macular edema, Uveitis, Panuveitis, Pediatric uveitis

## Abstract

**Background:**

Macular edema is a common complication of uveitis and represents a therapeutic challenge, especially in children. Recently, intravitreal dexamethasone implants have been shown to decrease intraocular inflammation and to control uveitic macular edema in patients with non-infectious intermediate or posterior uveitis.

**Findings:**

An 11-year-old boy with bilateral granulomatous idiopathic panuveitis and orbital inflammation experienced macular edema refractory to topical steroids and subcutaneous methotrexate. He was treated with off-label bilateral injections of dexamethasone intravitreal implant. Three months later, his vision had improved from 20/200 in both eyes to 20/30 in the right eye and 20/40 in the left eye. Optical coherence tomography showed complete resolution of the cystoid macular edema and subretinal fluid in both eyes.

**Conclusions:**

This is a rare report of the use of bilateral dexamethasone intravitreal implant in a pediatric patient. The implants achieved complete resolution of the uveitic macular edema with no adverse events 3 months post-implantation.

## Findings

### Introduction

The prevalence of pediatric cases within the general uveitis population is estimated to range from 5% to 13.8% [1-3]. The treatment of uveitis in children is challenging due to the lack of verbalization of symptoms, difficult examination, and suboptimal compliance to the recommended treatment [4]. Corticosteroids remain the mainstay of treatment but are associated with unique complications in children especially when used systemically, such as growth retardation [5-7]. Intravitreal triamcinolone has been used to treat uveitic macular edema but is associated with a significant risk of increased intraocular pressure and cataract [8,9]. A novel dexamethasone intravitreal implant (Ozurdex^®^, Allergan Inc., Irvine, CA, USA) has been shown to significantly improve intraocular inflammation, visual acuity, and central macular thickness in adults with non-infectious intermediate or posterior uveitis [10]. Recently, control of inflammation and resolution of cystoid macular edema (CME) was achieved with dexamethasone implant in 13 out of 14 eyes of patients with pediatric uveitis [11]. We present a rare case of a child followed for bilateral recalcitrant uveitic macular edema despite systemic immunosuppression who responded to dexamethasone intravitreal implants.

### Case report

**Figure 1:**
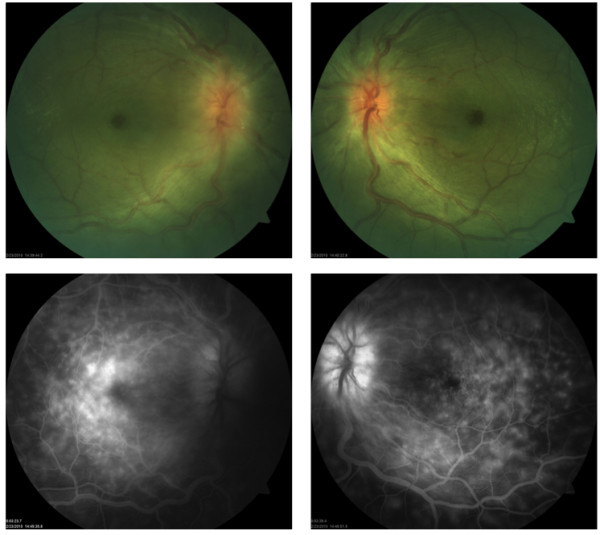
**Color fundus photographs and fluorescein angiogram at presentation.** (top right and top left) Both eyes showed optic nerve edema, dilated veins, and macular edema. (bottom right and bottom left) Late-phase fluorescein angiogram showed leakage of the optic nerve head that was more evident in the left eye, as well as patchy hyperfluorescence of the macula and petaloid leakage at the fovea in both eyes.

An 11-year-old boy of Nigerian descent was followed for bilateral granulomatous idiopathic panuveitis and orbital inflammation for 14 months (Figure 1). His past medical history was significant for hemoglobin SC disease and G6PD deficiency. At the onset of the disease, a rheumatologic workup with anti-nuclear antibody, rheumatoid factor, anti-neutrophil cytoplasmic antibodies, and HLA-B27 testing was negative. Serology for Lyme disease, toxoplasmosis, toxacariasis, cat scratch disease, human immunodeficiency virus, leptospirosis, coccidioidomycosis, and histoplasmosis was negative. Syphilis was also ruled out by a negative RPR and TP-PA. His serum calcium level was slightly elevated, but the angiotensin converting enzyme level and chest X-ray were normal. A biopsy of an enlarged right lacrimal gland biopsy was performed. Pathologic analysis was inconclusive and showed a non-specific non-granulomatous lymphocytic inflammation. A Mantoux test result showed 4 mm of induration and was considered positive as the patient came from an endemic area and was at that time treated with high-dose intravenous corticosteroids for his ocular inflammation. The patient was treated with anti-tuberculous triple-drug therapy for 2 months and then with rifampin for 4 months. Triple-drug therapy was discontinued as the patient was diagnosed with latent tuberculosis by infectious disease specialists. Indeed, he showed a positive response to corticosteroids, and aqueous and vitreous fluid cultures were negative for mycobacteria.

The patient had undergone a diagnostic vitrectomy with intravitreal injection of triamcinolone acetonide in the left eye 3 months after presentation for worsening of macular edema despite treatment with subcutaneous methotrexate for 6 weeks. The vitreous biopsy yielded negative cultures for bacteria, mycobacteria, fungus, and parasites. The central foveal thickness dramatically decreased from 1,002 μm preoperatively to 238 μm with complete resolution of the uveitic macular edema at 1 month after surgery.

**Figure 2:**
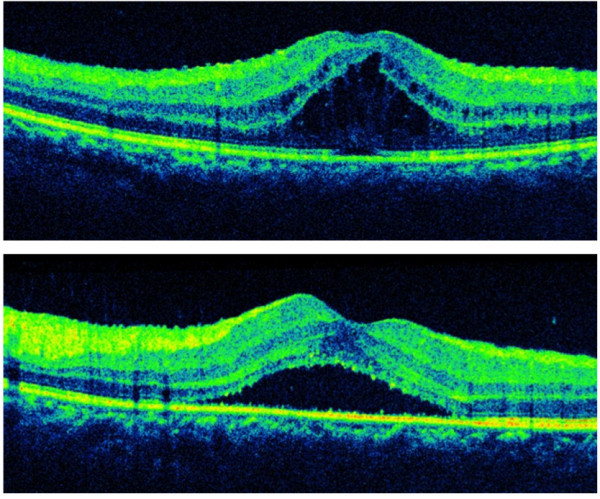
**Optical coherence tomography before bilateral injections of dexamethasone intravitreal implant.** Optical coherence tomography showed marked cystoid macular edema in the right eye. (top) The left eye showed subretinal fluid and minimal cystoid macular edema (bottom). The central retinal thickness was 592 μm in the right eye and 435 μm in the left eye.

Over the last 3 months, his best-corrected visual acuity had decreased from 20/40 in the right eye (OD) and 20/30 in the left eye (OS) to 20/200 in both eyes (OU). Slit lamp examination showed mutton-fat keratic precipitates, 4+ anterior chamber cells, and 3+ flare. Both eyes showed 4+ vitreous cells. Dilated fundus examination showed bilateral disc edema, dilated and sheathed veins, and macular edema. Optical coherence tomography (OCT) showed CME and subretinal fluid with a central foveal thickness of 592 μm OD and 435 μm OS (Figure 2). His treatment regimen included prednisolone drops 1% every hour, dexamethasone ointment 0.1% and homatropine drops 2% bid OU, subcutaneous methotrexate 20 mg weekly, and oral folic acid 2 mg daily.

**Figure 3:**
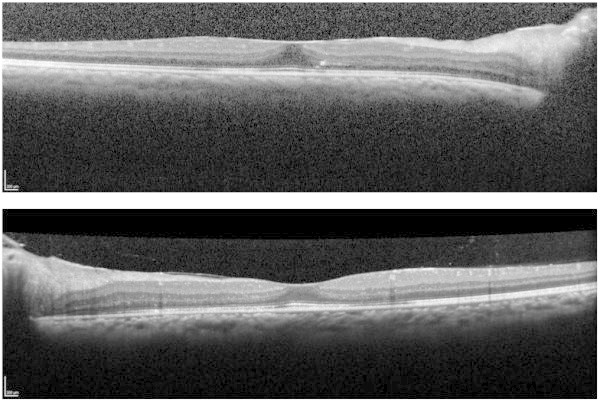
**Optical coherence tomography 3 months after bilateral injections of dexamethasone intravitreal implant.** Both eyes showed absence of cystoid macular edema and subretinal fluid (OD, top; OS, bottom). The central retinal thickness was 338 μm in the right eye and 261 μm in the left eye.

Given the refractory nature of the uveitic macular edema despite therapeutic levels of methotrexate, the prior positive response to intravitreal steroid and the vitrectomized status of the left eye, off-label bilateral injections of dexamethasone intravitreal implant were performed under general anesthesia. On post-operative day 1, the methotrexate was substituted for oral mycophenolate mofetil 500 mg daily, which was gradually increased to 750 mg bid. His visual acuity improved to 20/80 OD and 20/50 OS 3 weeks after the injections. Three months post-operatively, his visual acuity was 20/30 OD and 20/40 OS. He showed fine keratic precipitates, 2+ anterior chamber cells, and 1+ vitreous cells OU. The intraocular pressures were in the normal range and the both eyes showed posterior subcapsular cataracts. Both optic nerves showed reduced edema. The OCT showed absence of CME and subretinal fluid in both eyes (Figure 3).

The patient did not experience significant uveitic macular edema recurrence requiring local treatment over the next 2 years. Further systemic therapy with infliximab, which was added 11 months after the Ozurdex injections, has achieved excellent control of his uveitis.

### Discussion

CME is a common complication of pediatric uveitis with a prevalence of approximately 30% at 3 years from initial presentation [3,6]. In a large case series of 527 pediatric uveitis patients, CME was second only to hypotony as the uveitic complication with the most significant visual impact [6]. Our patient demonstrated macular edema that was refractory to topical steroids and immunosuppression with methotrexate but that was exquisitely responsive to vitrectomy and intravitreal triamcinolone acetonide in one eye.

When he experienced a second flare-up of his uveitic macular edema, the newly available Ozurdex^®^ implant was considered because of its duration of action and its stability in vitrectomized eyes. The half-life of intravitreal triamcinolone acetonide is shorter in a vitrectomized eye, its concentration decreasing 1.5 times more rapidly than in a non-vitrectomized eye [12]. On the other hand, Ozurdex^®^ exhibits a vitreous concentration of dexamethasone that is sustained over time to a similar extent in vitrectomized and non-vitrectomized eyes [13]. Moreover, pharmacokinetic and pharmacodynamic data suggest that when injected into the posterior segment, the implant releases dexamethasone into the vitreoretinal tissues for up to 6 months [14]. Three months after the injections, our patient experienced a significant visual acuity improvement and a normalization of his OCT in both eyes. He did not show increased intraocular pressure but posterior subcapsular cataracts started to develop. The methotrexate treatment was replaced by mycophenolate mofetil around the same time that the implants were injected and it may have assisted in the macular edema control. Mycophenolate mofetil takes several weeks to have an effect and probably did not play a significant role in the initial control of the macular edema. However, it possibly helped to keep the macula dry after the effect of the implants wore off.

This case did not only represent a therapeutic challenge, but also a diagnostic one. Tuberculosis was first considered as the etiology of our patient’s uveitis but that hypothesis was later abandoned. Although a strong suspicion for sarcoid-associated uveitis and orbital disease was raised, this diagnosis could not be confirmed despite an extensive work-up including a biopsy of an enlarged lacrimal gland. The etiology was therefore labeled as idiopathic.

In a previous report of the use of the Ozurdex^®^ intravitreal implant for the treatment of uveitis in children, 31% of treated eyes that showed an initial response relapsed within 6 months of their injection, at a median time of 4 months [11]. This finding stresses the importance of concomitant systemic immunosuppressive therapy to achieve long-term control of ocular inflammation. Lowder et al. showed that dexamethasone intravitreal implant was effective in controlling ocular inflammation and had an advantageous safety profile, with less than 10% of treated eyes having an intraocular pressure of 25 mmHg or greater and no significant increased risk of cataract [10]. Nevertheless, they did not study the safety profile of repeated injections. We believe that Ozurdex^®^ has a role in the treatment of pediatric uveitis or its complications, such as macular edema, which are not well controlled with systemic therapy or when compliance with medication may be a problem.

### Consent

Written informed consent was obtained from the patient’s guardian/parent/next of kin for the publication of this report and any accompanying images.

## Abbreviations

CME: Cystoid macular edema; OD: Right eye; OS: Left eye; OU: Both eyes; OCT: Optical coherence tomography.

## Competing interests

The authors declare that they have no competing interests.

## Authors’ contributions

SB performed a literature review and drafted the manuscript. AL drafted the manuscript. MA, LAW, and PEM managed the case and revised the manuscript critically. All authors read and approved the final manuscript.
